# 2,9,12,15,18,25,27,34,37,40,43,50-Dodeca­oxa-56-aza­hepta­cyclo­[24.24.5.1^51,55^.0^3,8^.0^19,24^.0^28,33^.0^44,49^]hexa­penta­conta-3,5,7,19(24),20,22,28(33),29,31,44,46,48,51,53,55-penta­deca­ene

**DOI:** 10.1107/S1600536812038093

**Published:** 2012-09-12

**Authors:** Jun Hee Kim, Il Yoon, Wonbo Sim, Jai Young Lee

**Affiliations:** a Department of Chemistry, Konyang University, Nonsan 320-711, Republic of Korea; b PDT Research Institute, School of Nano System Engineering, Inje University, Gimhae 621-749, Republic of Korea

## Abstract

The title compound, C_43_H_45_NO_12_, was prepared from the reaction of 2,6-bis­(dibromo­meth­yl)pyridine and bis­phenol in the presence of caesium carbonate as a base. The central ring makes dihedral angles of 64.83 (6), 13.48 (6), 56.96 (6) and 66.21 (6)° with the peripheral rings. In the crystal, mol­ecules are linked by weak C—H⋯O and C—H⋯π inter­actions, forming a folded structure.

## Related literature

For background to crown ether-based macrocyclic compounds and their inclusion behaviour, see: Weber & Vögtle, (1976 ▶, 1980 ▶). For the preparation and crystal structures of related compounds, see: Lee *et al.* (2009 ▶); Beack *et al.* (2012 ▶).
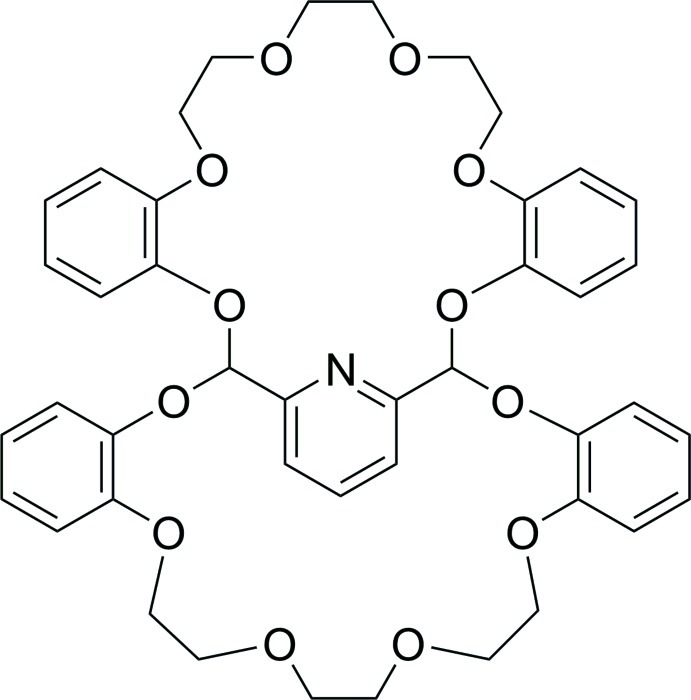


## Experimental

### 

#### Crystal data

C_43_H_45_NO_12_
*M* *_r_* = 767.80
Triclinic, 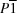

*a* = 8.5266 (4) Å
*b* = 15.2732 (7) Å
*c* = 15.5688 (8) Å
α = 69.752 (2)°
β = 88.372 (2)°
γ = 80.402 (2)°
*V* = 1874.64 (16) Å^3^
*Z* = 2
Mo *K*α radiation
μ = 0.10 mm^−1^
*T* = 296 K
0.25 × 0.19 × 0.13 mm

#### Data collection

Bruker APEXII CCD diffractometer
Absorption correction: multi-scan (*SADABS*; Sheldrick, 1996 ▶) *T*_min_ = 0.975, *T*_max_ = 0.988
33651 measured reflections
8622 independent reflections
7657 reflections with *I* > 2σ(*I*)
*R*_int_ = 0.024

#### Refinement

*R*[*F* ^2^ > 2σ(*F* ^2^)] = 0.037
*wR*(*F* ^2^) = 0.097
*S* = 1.03
8622 reflections
505 parameters
H-atom parameters constrained
Δρ_max_ = 0.28 e Å^−3^
Δρ_min_ = −0.27 e Å^−3^

### 

Data collection: *APEX2* (Bruker, 2000 ▶); cell refinement: *SAINT-Plus* (Bruker, 2000 ▶); data reduction: *SAINT-Plus*; program(s) used to solve structure: *SHELXTL* (Sheldrick, 2008 ▶); program(s) used to refine structure: *SHELXTL*; molecular graphics: *SHELXTL*; software used to prepare material for publication: *SHELXTL*

## Supplementary Material

Crystal structure: contains datablock(s) I, global. DOI: 10.1107/S1600536812038093/lx2264sup1.cif

Structure factors: contains datablock(s) I. DOI: 10.1107/S1600536812038093/lx2264Isup2.hkl

Additional supplementary materials:  crystallographic information; 3D view; checkCIF report

## Figures and Tables

**Table 1:** Hydrogen-bond geometry (Å, °) *Cg*1 and *Cg*2 are the centroids of the C7–C12 benzene ring and the N1/C1–C5 pyridine ring, respectively.

*D*—H⋯*A*	*D*—H	H⋯*A*	*D*⋯*A*	*D*—H⋯*A*
C8—H8*A*⋯O4^i^	0.93	2.44	3.2871 (14)	151
C17—H17*A*⋯O11^ii^	0.97	2.60	3.4207 (15)	142
C3—H3*A*⋯*Cg*1^i^	0.93	2.94	3.6031 (15)	130
C18—H18*B*⋯*Cg*2^i^	0.97	2.80	3.7531 (15)	167

Symmetry codes: (i) 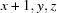; (ii) 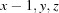.

**Fig. 1.**
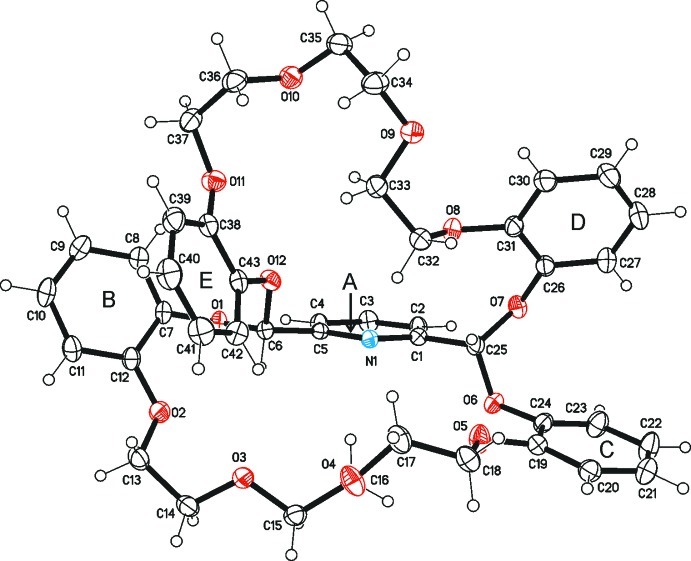
The molecular structure of the title compound with the atom numbering scheme and intramolecular C—H···O and π···π interactions. Displacement ellipsoids are drawn at the 30% probability level. H atoms non-participating in hydrogen-bonding were omitted for clarity.

**Fig. 2.**
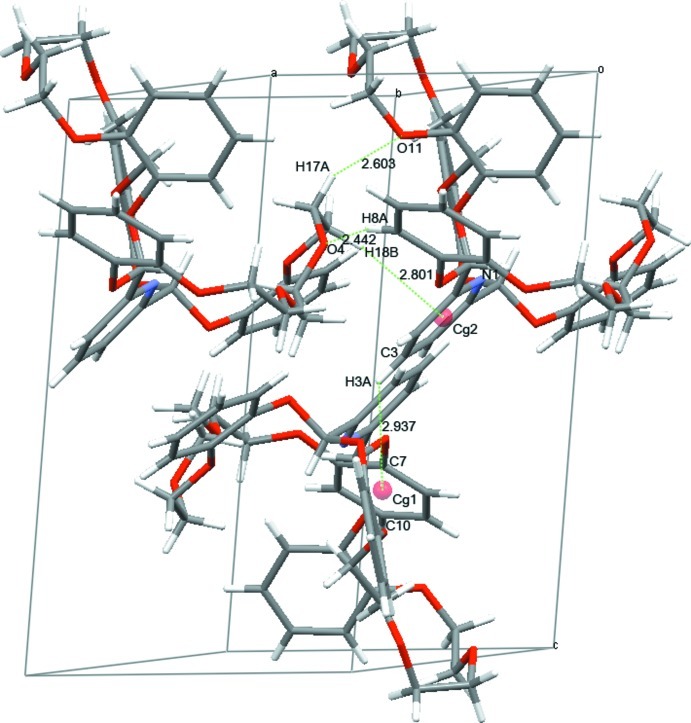
A view of the C—H···O and C—H···π interactions (dotted lines) in the crystal structure of the title compound. H atoms non-participating in hydrogen-bonding were omitted for clarity. [Symmetry codes: (i) x+1, y, z ; (ii) x-1, y, z.]

## supplementary crystallographic information

### Comment

In our previous paper (Lee *et al.*, 2009, Beack *et al.*, 2012), we synthesized and reported the preparation and the solid-state structure of new crown ether bearing three aromatic subunits. As a part of our continuing interest in the development of new crown compounds, the preparation and crystal structure of new crown ether-based macrocyclic compound containing pyridine unit (Weber *et al.*, 1976, 1980), we report herein the crystal structure of the title compound.

The crystal structure exhibits a twisted conformation with dihedral angles of 64.83 (6) (A/B), 13.48 (6) (A/C), 56.96 (6) (A/D), 66.21 (6) (A/E), 35.31 (6) (B/E), 69.15 (6) (C/D), 80.92 (6)° (D/E). (Fig. 1. A is N1/C1-C5 pyridine ring, B, C, D, E, are C7–C12, C19–C24, C26–C31 and C38–C43 benzene rings, respectively. In the crystal, molecules are linked by weak intermolecular C—H···O and C—H···π interactions. (Fig. 2 & Table 1; Cg1 and Cg2 are the centroids of the C7–C12 benzene ring and the N1/C1–C5 pyridine ring, respectively).

### Experimental

To a refluxing suspension of caesium carbonate (14.1 mmol) in THF under nitrogen was added dropwise a solution of 2,6-bis(dibromomethyl)pyridine (2.82 mmol) and 1,8-bis(2-hydroxyphenoxy)-3,6-dioxaoctane (6.21 mmol) in THF over a period of 1 h. The mixture was then refluxed for an additional 48 h. After cooling to room temperature, 10% aqueous hydrochloric acid was added. The solvent was removed under reduced pressure and the residual mixture was extracted with dichloromethane. The organic layer was washed with water, dried over anhydrous magnesium sulfate, and evaporated *in vacuo*. The crude product was chromatographed on a silica-gel column using a mixed solvent of ethyl acetate and *n*-hexane (1:2) as eluent, and recrystallization from dichloromethane/n-hexane (1:30, *v*/*v*) gave as a crystalline solid in 24% yield (m.p. 402–404 K).

### Refinement

All H atoms were positioned geometrically and refined using a riding model, with C—H = 0.93 Å for aryl, 0.98 Å for methine, and 0.97 Å for methylene H atoms. *U*_iso_(H) = 1.2*U*_eq_(C) for all H atoms.

### Crystal data

**Table:**

C_43_H_45_NO_12_	*Z* = 2
*M**_r_* = 767.80	*F*(000) = 812
Triclinic, *P*1	*D*_x_ = 1.360 Mg m^−^^3^
Hall symbol: -P 1	Mo *K*α radiation, λ = 0.71073 Å
*a* = 8.5266 (4) Å	Cell parameters from 9368 reflections
*b* = 15.2732 (7) Å	θ = 2.6–27.5°
*c* = 15.5688 (8) Å	µ = 0.10 mm^−^^1^
α = 69.752 (2)°	*T* = 296 K
β = 88.372 (2)°	Block, colourless
γ = 80.402 (2)°	0.25 × 0.19 × 0.13 mm
*V* = 1874.64 (16) Å^3^	

### Data collection

**Table:**

Bruker APEXII CCD diffractometer	8622 independent reflections
Radiation source: fine-focus sealed tube	7657 reflections with *I* > 2σ(*I*)
Graphite monochromator	*R*_int_ = 0.024
π and ω scans	θ_max_ = 27.6°, θ_min_ = 1.4°
Absorption correction: multi-scan (*SADABS*; Sheldrick, 1996)	*h* = −11→11
*T*_min_ = 0.975, *T*_max_ = 0.988	*k* = −19→19
33651 measured reflections	*l* = −20→20

### Refinement

**Table:**

Refinement on *F*^2^	Primary atom site location: structure-invariant direct methods
Least-squares matrix: full	Secondary atom site location: difference Fourier map
*R*[*F*^2^ > 2σ(*F*^2^)] = 0.037	Hydrogen site location: difference Fourier map
*wR*(*F*^2^) = 0.097	H-atom parameters constrained
*S* = 1.03	*w* = 1/[σ^2^(*F*_o_^2^) + (0.0469*P*)^2^ + 0.7124*P*] where *P* = (*F*_o_^2^ + 2*F*_c_^2^)/3
8622 reflections	(Δ/σ)_max_ = 0.001
505 parameters	Δρ_max_ = 0.28 e Å^−^^3^
0 restraints	Δρ_min_ = −0.27 e Å^−^^3^

### Special details

**Table:**

Experimental. IR(KBr pellet, cm^-1^): 2926, 1583, 1500, 1454, 1260, 1122, 1055, 973, 745. ^1^H NMR (CDCl_3_): δ 7.98 (d, 2 H, pyd-*H*), 7.93 (t, 1 H, pyd-*H*). 7.06~6.72 (m, 16 H, Ar-*H*), 6.86 (s, 2 H, pyd-C*H*OO), 3.95(t, 8 H, ArOC*H*_2_CH_2_O), 3.58(t, 8 H, ArOCH_2_C*H*_2_O), 3.39(s, 8 H, ArOCH_2_CH_2_OC*H*_2_C*H*_2_O). ^13^C NMR (CDCl_3_): d 157.443, 151.085, 146.497, 137.688, 124.763, 122.382, 121.690, 114.131, 104.316, 71.976, 70.049, 69.576 p.p.m..
Geometry. All esds (except the esd in the dihedral angle between two l.s. planes) are estimated using the full covariance matrix. The cell esds are taken into account individually in the estimation of esds in distances, angles and torsion angles; correlations between esds in cell parameters are only used when they are defined by crystal symmetry. An approximate (isotropic) treatment of cell esds is used for estimating esds involving l.s. planes.
Refinement. Refinement of F^2^ against ALL reflections. The weighted R-factor wR and goodness of fit S are based on F^2^, conventional R-factors R are based on F, with F set to zero for negative F^2^. The threshold expression of F^2^ > 2sigma(F^2^) is used only for calculating R-factors(gt) etc. and is not relevant to the choice of reflections for refinement. R-factors based on F^2^ are statistically about twice as large as those based on F, and R- factors based on ALL data will be even larger.

### Fractional atomic coordinates and isotropic or equivalent isotropic displacement parameters (Å^2^)

**Table:**

	*x*	*y*	*z*	*U*_iso_*/*U*_eq_	
N1	0.16048 (10)	0.23963 (6)	0.34935 (6)	0.01732 (18)	
O1	0.42156 (9)	0.02455 (5)	0.36551 (5)	0.02018 (16)	
O2	0.23385 (10)	−0.10515 (6)	0.38118 (6)	0.02562 (18)	
O3	−0.06713 (10)	−0.01380 (6)	0.37368 (6)	0.02620 (18)	
O4	−0.29589 (11)	0.15178 (6)	0.28839 (6)	0.0302 (2)	
O5	−0.30106 (10)	0.34100 (6)	0.31315 (6)	0.02827 (19)	
O6	−0.09806 (9)	0.37352 (6)	0.42255 (5)	0.02140 (16)	
O7	0.06804 (10)	0.47547 (5)	0.34239 (6)	0.02325 (17)	
O8	0.18306 (10)	0.43854 (6)	0.18692 (6)	0.02400 (17)	
O9	0.25086 (11)	0.45435 (6)	−0.00716 (6)	0.02920 (19)	
O10	0.55737 (10)	0.32192 (6)	−0.01372 (6)	0.02566 (18)	
O11	0.49707 (10)	0.14304 (6)	0.10207 (6)	0.02586 (18)	
O12	0.31057 (10)	0.14756 (5)	0.23457 (5)	0.02138 (16)	
C1	0.15225 (12)	0.30759 (7)	0.38556 (7)	0.0179 (2)	
C2	0.26197 (14)	0.30632 (8)	0.45049 (8)	0.0221 (2)	
H2A	0.2531	0.3556	0.4734	0.027*	
C3	0.38433 (14)	0.23023 (8)	0.48010 (8)	0.0234 (2)	
H3A	0.4587	0.2270	0.5241	0.028*	
C4	0.39516 (13)	0.15860 (7)	0.44349 (7)	0.0195 (2)	
H4A	0.4759	0.1063	0.4627	0.023*	
C5	0.28230 (12)	0.16721 (7)	0.37739 (7)	0.0167 (2)	
C6	0.28892 (12)	0.09688 (7)	0.32825 (7)	0.0173 (2)	
H6A	0.1903	0.0703	0.3359	0.021*	
C7	0.46007 (13)	−0.04066 (7)	0.32113 (7)	0.0194 (2)	
C8	0.59910 (14)	−0.04026 (8)	0.27378 (8)	0.0236 (2)	
H8A	0.6615	0.0058	0.2682	0.028*	
C9	0.64556 (15)	−0.10925 (9)	0.23426 (9)	0.0296 (3)	
H9A	0.7401	−0.1100	0.2031	0.035*	
C10	0.55131 (16)	−0.17632 (9)	0.24142 (9)	0.0308 (3)	
H10A	0.5829	−0.2222	0.2150	0.037*	
C11	0.40934 (15)	−0.17611 (8)	0.28783 (9)	0.0267 (2)	
H11A	0.3452	−0.2208	0.2912	0.032*	
C12	0.36402 (13)	−0.10881 (8)	0.32907 (8)	0.0212 (2)	
C13	0.10545 (14)	−0.14958 (8)	0.36882 (9)	0.0273 (2)	
H13A	0.1333	−0.2179	0.3959	0.033*	
H13B	0.0803	−0.1330	0.3042	0.033*	
C14	−0.03361 (14)	−0.11372 (8)	0.41581 (9)	0.0266 (2)	
H14A	−0.1254	−0.1413	0.4097	0.032*	
H14B	−0.0081	−0.1308	0.4805	0.032*	
C15	−0.17984 (14)	0.03039 (8)	0.42142 (8)	0.0243 (2)	
H15A	−0.1398	0.0171	0.4831	0.029*	
H15B	−0.2791	0.0063	0.4253	0.029*	
C16	−0.20694 (14)	0.13527 (8)	0.36989 (8)	0.0239 (2)	
H16A	−0.2653	0.1687	0.4068	0.029*	
H16B	−0.1059	0.1574	0.3551	0.029*	
C17	−0.31024 (16)	0.24439 (9)	0.22326 (8)	0.0288 (3)	
H17A	−0.3607	0.2455	0.1677	0.035*	
H17B	−0.2044	0.2593	0.2082	0.035*	
C18	−0.40408 (15)	0.32034 (8)	0.25400 (8)	0.0258 (2)	
H18A	−0.4397	0.3767	0.2015	0.031*	
H18B	−0.4967	0.2985	0.2866	0.031*	
C19	−0.35332 (13)	0.41479 (8)	0.34239 (8)	0.0212 (2)	
C20	−0.50285 (14)	0.47113 (8)	0.32230 (8)	0.0245 (2)	
H20A	−0.5782	0.4579	0.2885	0.029*	
C21	−0.53898 (15)	0.54706 (9)	0.35292 (9)	0.0307 (3)	
H21A	−0.6386	0.5851	0.3388	0.037*	
C22	−0.42956 (16)	0.56692 (10)	0.40388 (10)	0.0355 (3)	
H22A	−0.4545	0.6187	0.4232	0.043*	
C23	−0.28131 (15)	0.50912 (9)	0.42638 (9)	0.0295 (3)	
H23A	−0.2077	0.5215	0.4618	0.035*	
C24	−0.24379 (13)	0.43376 (8)	0.39619 (8)	0.0214 (2)	
C25	0.01485 (13)	0.38871 (7)	0.35244 (7)	0.0189 (2)	
H25A	−0.0319	0.3911	0.2947	0.023*	
C26	0.05292 (13)	0.54533 (8)	0.25695 (8)	0.0231 (2)	
C27	−0.01090 (16)	0.63690 (9)	0.25206 (10)	0.0325 (3)	
H27A	−0.0470	0.6487	0.3046	0.039*	
C28	−0.02063 (18)	0.71077 (9)	0.16855 (11)	0.0393 (3)	
H28A	−0.0623	0.7721	0.1654	0.047*	
C29	0.03125 (16)	0.69367 (9)	0.09012 (10)	0.0350 (3)	
H29A	0.0227	0.7432	0.0342	0.042*	
C30	0.09607 (14)	0.60270 (9)	0.09464 (9)	0.0283 (3)	
H30A	0.1313	0.5913	0.0418	0.034*	
C31	0.10854 (13)	0.52837 (8)	0.17819 (8)	0.0227 (2)	
C32	0.11733 (14)	0.39272 (8)	0.13235 (8)	0.0239 (2)	
H32A	0.0240	0.4341	0.0980	0.029*	
H32B	0.0843	0.3349	0.1726	0.029*	
C33	0.23586 (15)	0.36951 (8)	0.06713 (8)	0.0254 (2)	
H33A	0.3382	0.3412	0.0986	0.030*	
H33B	0.2007	0.3244	0.0440	0.030*	
C34	0.32053 (15)	0.43884 (9)	−0.08583 (8)	0.0288 (3)	
H34A	0.2906	0.4956	−0.1389	0.035*	
H34B	0.2776	0.3881	−0.0958	0.035*	
C35	0.50017 (15)	0.41369 (9)	−0.07829 (9)	0.0282 (3)	
H35A	0.5403	0.4168	−0.1380	0.034*	
H35B	0.5423	0.4605	−0.0607	0.034*	
C36	0.54982 (16)	0.24892 (9)	−0.04898 (8)	0.0286 (3)	
H36A	0.6199	0.2547	−0.1001	0.034*	
H36B	0.4421	0.2531	−0.0704	0.034*	
C37	0.60036 (15)	0.15579 (9)	0.02652 (9)	0.0289 (3)	
H37A	0.6010	0.1048	0.0028	0.035*	
H37B	0.7079	0.1527	0.0475	0.035*	
C38	0.35459 (14)	0.11590 (8)	0.09707 (8)	0.0215 (2)	
C39	0.30404 (15)	0.08776 (9)	0.02814 (8)	0.0272 (2)	
H39A	0.3683	0.0885	−0.0213	0.033*	
C40	0.15708 (16)	0.05847 (9)	0.03322 (9)	0.0305 (3)	
H40A	0.1244	0.0385	−0.0124	0.037*	
C41	0.05942 (15)	0.05870 (9)	0.10527 (9)	0.0294 (3)	
H41A	−0.0385	0.0388	0.1081	0.035*	
C42	0.10706 (14)	0.08869 (8)	0.17370 (8)	0.0246 (2)	
H42A	0.0400	0.0906	0.2214	0.029*	
C43	0.25443 (13)	0.11560 (7)	0.17044 (7)	0.0200 (2)	

### Atomic displacement parameters (Å^2^)

**Table:**

	*U*^11^	*U*^22^	*U*^33^	*U*^12^	*U*^13^	*U*^23^
N1	0.0178 (4)	0.0167 (4)	0.0183 (4)	−0.0034 (3)	0.0012 (3)	−0.0068 (3)
O1	0.0203 (4)	0.0186 (4)	0.0234 (4)	0.0012 (3)	−0.0028 (3)	−0.0113 (3)
O2	0.0233 (4)	0.0263 (4)	0.0326 (4)	−0.0074 (3)	0.0036 (3)	−0.0156 (4)
O3	0.0253 (4)	0.0204 (4)	0.0288 (4)	−0.0022 (3)	0.0060 (3)	−0.0046 (3)
O4	0.0370 (5)	0.0203 (4)	0.0340 (5)	−0.0065 (3)	−0.0120 (4)	−0.0083 (4)
O5	0.0252 (4)	0.0253 (4)	0.0392 (5)	0.0001 (3)	−0.0074 (4)	−0.0187 (4)
O6	0.0182 (4)	0.0241 (4)	0.0211 (4)	0.0004 (3)	0.0008 (3)	−0.0086 (3)
O7	0.0264 (4)	0.0181 (4)	0.0267 (4)	−0.0032 (3)	−0.0016 (3)	−0.0096 (3)
O8	0.0226 (4)	0.0212 (4)	0.0273 (4)	0.0023 (3)	−0.0012 (3)	−0.0099 (3)
O9	0.0335 (5)	0.0239 (4)	0.0270 (4)	−0.0037 (3)	0.0087 (3)	−0.0060 (3)
O10	0.0277 (4)	0.0261 (4)	0.0241 (4)	−0.0056 (3)	0.0015 (3)	−0.0094 (3)
O11	0.0278 (4)	0.0291 (4)	0.0229 (4)	−0.0090 (3)	0.0024 (3)	−0.0101 (3)
O12	0.0294 (4)	0.0203 (4)	0.0171 (4)	−0.0079 (3)	0.0007 (3)	−0.0081 (3)
C1	0.0178 (5)	0.0172 (5)	0.0193 (5)	−0.0035 (4)	0.0019 (4)	−0.0067 (4)
C2	0.0261 (6)	0.0200 (5)	0.0230 (5)	−0.0026 (4)	−0.0017 (4)	−0.0115 (4)
C3	0.0249 (6)	0.0238 (5)	0.0228 (5)	−0.0027 (4)	−0.0056 (4)	−0.0099 (4)
C4	0.0192 (5)	0.0183 (5)	0.0200 (5)	−0.0007 (4)	−0.0011 (4)	−0.0065 (4)
C5	0.0181 (5)	0.0160 (5)	0.0164 (5)	−0.0045 (4)	0.0031 (4)	−0.0055 (4)
C6	0.0178 (5)	0.0158 (5)	0.0178 (5)	−0.0017 (4)	−0.0004 (4)	−0.0057 (4)
C7	0.0217 (5)	0.0170 (5)	0.0195 (5)	0.0019 (4)	−0.0044 (4)	−0.0084 (4)
C8	0.0231 (5)	0.0232 (5)	0.0252 (6)	−0.0017 (4)	−0.0014 (4)	−0.0102 (4)
C9	0.0268 (6)	0.0339 (6)	0.0312 (6)	−0.0004 (5)	0.0040 (5)	−0.0177 (5)
C10	0.0341 (7)	0.0299 (6)	0.0347 (7)	0.0017 (5)	−0.0007 (5)	−0.0221 (5)
C11	0.0291 (6)	0.0223 (5)	0.0327 (6)	−0.0029 (4)	−0.0032 (5)	−0.0146 (5)
C12	0.0209 (5)	0.0191 (5)	0.0227 (5)	0.0008 (4)	−0.0035 (4)	−0.0078 (4)
C13	0.0261 (6)	0.0208 (5)	0.0374 (7)	−0.0068 (4)	0.0002 (5)	−0.0118 (5)
C14	0.0257 (6)	0.0204 (5)	0.0321 (6)	−0.0073 (4)	0.0025 (5)	−0.0057 (5)
C15	0.0205 (5)	0.0255 (6)	0.0258 (6)	−0.0041 (4)	0.0022 (4)	−0.0077 (5)
C16	0.0216 (5)	0.0248 (6)	0.0269 (6)	−0.0052 (4)	−0.0010 (4)	−0.0102 (5)
C17	0.0387 (7)	0.0236 (6)	0.0251 (6)	−0.0061 (5)	−0.0033 (5)	−0.0089 (5)
C18	0.0281 (6)	0.0224 (5)	0.0273 (6)	−0.0063 (4)	−0.0061 (5)	−0.0076 (5)
C19	0.0221 (5)	0.0185 (5)	0.0228 (5)	−0.0039 (4)	0.0024 (4)	−0.0067 (4)
C20	0.0215 (5)	0.0243 (5)	0.0255 (6)	−0.0039 (4)	−0.0011 (4)	−0.0059 (4)
C21	0.0231 (6)	0.0298 (6)	0.0367 (7)	0.0043 (5)	−0.0004 (5)	−0.0121 (5)
C22	0.0314 (7)	0.0339 (7)	0.0469 (8)	0.0049 (5)	−0.0010 (6)	−0.0257 (6)
C23	0.0256 (6)	0.0341 (7)	0.0347 (7)	−0.0010 (5)	−0.0013 (5)	−0.0209 (5)
C24	0.0178 (5)	0.0227 (5)	0.0230 (5)	−0.0011 (4)	0.0023 (4)	−0.0083 (4)
C25	0.0192 (5)	0.0186 (5)	0.0205 (5)	−0.0026 (4)	0.0012 (4)	−0.0093 (4)
C26	0.0191 (5)	0.0185 (5)	0.0310 (6)	−0.0027 (4)	0.0009 (4)	−0.0077 (4)
C27	0.0341 (7)	0.0223 (6)	0.0408 (7)	−0.0005 (5)	0.0065 (5)	−0.0127 (5)
C28	0.0412 (8)	0.0176 (6)	0.0533 (9)	0.0008 (5)	0.0056 (6)	−0.0081 (6)
C29	0.0339 (7)	0.0217 (6)	0.0398 (7)	−0.0027 (5)	0.0029 (5)	0.0002 (5)
C30	0.0238 (6)	0.0252 (6)	0.0316 (6)	−0.0036 (5)	0.0034 (5)	−0.0050 (5)
C31	0.0164 (5)	0.0197 (5)	0.0307 (6)	−0.0012 (4)	0.0009 (4)	−0.0078 (4)
C32	0.0238 (5)	0.0227 (5)	0.0252 (6)	−0.0045 (4)	0.0015 (4)	−0.0080 (4)
C33	0.0287 (6)	0.0214 (5)	0.0236 (6)	−0.0019 (4)	0.0029 (4)	−0.0060 (4)
C34	0.0285 (6)	0.0325 (6)	0.0221 (6)	−0.0050 (5)	0.0032 (5)	−0.0054 (5)
C35	0.0281 (6)	0.0282 (6)	0.0261 (6)	−0.0076 (5)	0.0044 (5)	−0.0058 (5)
C36	0.0319 (6)	0.0337 (6)	0.0246 (6)	−0.0094 (5)	0.0075 (5)	−0.0144 (5)
C37	0.0263 (6)	0.0300 (6)	0.0330 (6)	−0.0032 (5)	0.0069 (5)	−0.0151 (5)
C38	0.0254 (5)	0.0173 (5)	0.0212 (5)	−0.0020 (4)	−0.0018 (4)	−0.0064 (4)
C39	0.0347 (6)	0.0267 (6)	0.0232 (6)	−0.0043 (5)	0.0014 (5)	−0.0128 (5)
C40	0.0378 (7)	0.0314 (6)	0.0280 (6)	−0.0057 (5)	−0.0067 (5)	−0.0168 (5)
C41	0.0276 (6)	0.0310 (6)	0.0322 (6)	−0.0061 (5)	−0.0061 (5)	−0.0130 (5)
C42	0.0241 (6)	0.0258 (6)	0.0241 (5)	−0.0022 (4)	−0.0012 (4)	−0.0098 (5)
C43	0.0264 (5)	0.0153 (5)	0.0183 (5)	−0.0009 (4)	−0.0040 (4)	−0.0067 (4)

### Geometric parameters (Å, º)

**Table:**

N1—C1	1.3336 (13)	C16—H16A	0.9700
N1—C5	1.3421 (13)	C16—H16B	0.9700
O1—C7	1.3900 (12)	C17—C18	1.5060 (17)
O1—C6	1.4188 (12)	C17—H17A	0.9700
O2—C12	1.3612 (14)	C17—H17B	0.9700
O2—C13	1.4305 (14)	C18—H18A	0.9700
O3—C14	1.4189 (14)	C18—H18B	0.9700
O3—C15	1.4220 (14)	C19—C20	1.3908 (16)
O4—C17	1.4144 (14)	C19—C24	1.4002 (16)
O4—C16	1.4207 (14)	C20—C21	1.3864 (17)
O5—C19	1.3608 (13)	C20—H20A	0.9300
O5—C18	1.4349 (14)	C21—C22	1.3765 (19)
O6—C24	1.3947 (13)	C21—H21A	0.9300
O6—C25	1.4184 (13)	C22—C23	1.3924 (18)
O7—C26	1.3805 (14)	C22—H22A	0.9300
O7—C25	1.4279 (13)	C23—C24	1.3752 (16)
O8—C31	1.3746 (13)	C23—H23A	0.9300
O8—C32	1.4475 (14)	C25—H25A	0.9800
O9—C34	1.4219 (15)	C26—C27	1.3903 (16)
O9—C33	1.4292 (14)	C26—C31	1.3934 (17)
O10—C36	1.4136 (14)	C27—C28	1.389 (2)
O10—C35	1.4274 (15)	C27—H27A	0.9300
O11—C38	1.3599 (14)	C28—C29	1.381 (2)
O11—C37	1.4272 (14)	C28—H28A	0.9300
O12—C43	1.3807 (13)	C29—C30	1.3853 (18)
O12—C6	1.4182 (13)	C29—H29A	0.9300
C1—C2	1.3899 (15)	C30—C31	1.3920 (17)
C1—C25	1.5105 (14)	C30—H30A	0.9300
C2—C3	1.3798 (16)	C32—C33	1.4998 (16)
C2—H2A	0.9300	C32—H32A	0.9700
C3—C4	1.3874 (15)	C32—H32B	0.9700
C3—H3A	0.9300	C33—H33A	0.9700
C4—C5	1.3866 (15)	C33—H33B	0.9700
C4—H4A	0.9300	C34—C35	1.5133 (17)
C5—C6	1.5127 (14)	C34—H34A	0.9700
C6—H6A	0.9800	C34—H34B	0.9700
C7—C8	1.3779 (16)	C35—H35A	0.9700
C7—C12	1.3996 (16)	C35—H35B	0.9700
C8—C9	1.3935 (16)	C36—C37	1.5025 (18)
C8—H8A	0.9300	C36—H36A	0.9700
C9—C10	1.3777 (19)	C36—H36B	0.9700
C9—H9A	0.9300	C37—H37A	0.9700
C10—C11	1.3922 (18)	C37—H37B	0.9700
C10—H10A	0.9300	C38—C39	1.3890 (16)
C11—C12	1.3886 (15)	C38—C43	1.4059 (16)
C11—H11A	0.9300	C39—C40	1.3904 (19)
C13—C14	1.5009 (17)	C39—H39A	0.9300
C13—H13A	0.9700	C40—C41	1.3787 (19)
C13—H13B	0.9700	C40—H40A	0.9300
C14—H14A	0.9700	C41—C42	1.3908 (16)
C14—H14B	0.9700	C41—H41A	0.9300
C15—C16	1.5035 (16)	C42—C43	1.3801 (16)
C15—H15A	0.9700	C42—H42A	0.9300
C15—H15B	0.9700		
			
C1—N1—C5	117.46 (9)	C21—C20—H20A	120.2
C7—O1—C6	116.18 (8)	C19—C20—H20A	120.2
C12—O2—C13	118.30 (9)	C22—C21—C20	120.95 (11)
C14—O3—C15	113.10 (9)	C22—C21—H21A	119.5
C17—O4—C16	115.62 (9)	C20—C21—H21A	119.5
C19—O5—C18	118.56 (9)	C21—C22—C23	119.65 (12)
C24—O6—C25	113.49 (8)	C21—C22—H22A	120.2
C26—O7—C25	118.20 (9)	C23—C22—H22A	120.2
C31—O8—C32	116.75 (9)	C24—C23—C22	119.97 (12)
C34—O9—C33	114.12 (9)	C24—C23—H23A	120.0
C36—O10—C35	112.52 (9)	C22—C23—H23A	120.0
C38—O11—C37	119.56 (9)	C23—C24—O6	119.67 (10)
C43—O12—C6	117.59 (8)	C23—C24—C19	120.52 (10)
N1—C1—C2	123.34 (10)	O6—C24—C19	119.76 (10)
N1—C1—C25	116.51 (9)	O6—C25—O7	107.64 (8)
C2—C1—C25	120.15 (9)	O6—C25—C1	106.58 (8)
C3—C2—C1	118.40 (10)	O7—C25—C1	109.53 (9)
C3—C2—H2A	120.8	O6—C25—H25A	111.0
C1—C2—H2A	120.8	O7—C25—H25A	111.0
C2—C3—C4	119.27 (10)	C1—C25—H25A	111.0
C2—C3—H3A	120.4	O7—C26—C27	117.48 (11)
C4—C3—H3A	120.4	O7—C26—C31	122.60 (10)
C5—C4—C3	118.16 (10)	C27—C26—C31	119.77 (11)
C5—C4—H4A	120.9	C28—C27—C26	119.86 (13)
C3—C4—H4A	120.9	C28—C27—H27A	120.1
N1—C5—C4	123.32 (10)	C26—C27—H27A	120.1
N1—C5—C6	113.98 (9)	C29—C28—C27	120.39 (12)
C4—C5—C6	122.67 (9)	C29—C28—H28A	119.8
O12—C6—O1	110.70 (8)	C27—C28—H28A	119.8
O12—C6—C5	106.24 (8)	C28—C29—C30	120.03 (12)
O1—C6—C5	107.54 (8)	C28—C29—H29A	120.0
O12—C6—H6A	110.7	C30—C29—H29A	120.0
O1—C6—H6A	110.7	C29—C30—C31	120.06 (12)
C5—C6—H6A	110.7	C29—C30—H30A	120.0
C8—C7—O1	118.66 (10)	C31—C30—H30A	120.0
C8—C7—C12	120.82 (10)	O8—C31—C30	121.87 (11)
O1—C7—C12	120.42 (10)	O8—C31—C26	118.16 (10)
C7—C8—C9	119.56 (11)	C30—C31—C26	119.87 (11)
C7—C8—H8A	120.2	O8—C32—C33	111.66 (10)
C9—C8—H8A	120.2	O8—C32—H32A	109.3
C10—C9—C8	119.97 (11)	C33—C32—H32A	109.3
C10—C9—H9A	120.0	O8—C32—H32B	109.3
C8—C9—H9A	120.0	C33—C32—H32B	109.3
C9—C10—C11	120.73 (11)	H32A—C32—H32B	107.9
C9—C10—H10A	119.6	O9—C33—C32	109.20 (9)
C11—C10—H10A	119.6	O9—C33—H33A	109.8
C12—C11—C10	119.61 (11)	C32—C33—H33A	109.8
C12—C11—H11A	120.2	O9—C33—H33B	109.8
C10—C11—H11A	120.2	C32—C33—H33B	109.8
O2—C12—C11	125.04 (11)	H33A—C33—H33B	108.3
O2—C12—C7	115.61 (10)	O9—C34—C35	113.57 (10)
C11—C12—C7	119.30 (11)	O9—C34—H34A	108.9
O2—C13—C14	106.10 (9)	C35—C34—H34A	108.9
O2—C13—H13A	110.5	O9—C34—H34B	108.9
C14—C13—H13A	110.5	C35—C34—H34B	108.9
O2—C13—H13B	110.5	H34A—C34—H34B	107.7
C14—C13—H13B	110.5	O10—C35—C34	113.72 (10)
H13A—C13—H13B	108.7	O10—C35—H35A	108.8
O3—C14—C13	107.63 (9)	C34—C35—H35A	108.8
O3—C14—H14A	110.2	O10—C35—H35B	108.8
C13—C14—H14A	110.2	C34—C35—H35B	108.8
O3—C14—H14B	110.2	H35A—C35—H35B	107.7
C13—C14—H14B	110.2	O10—C36—C37	107.99 (10)
H14A—C14—H14B	108.5	O10—C36—H36A	110.1
O3—C15—C16	108.15 (9)	C37—C36—H36A	110.1
O3—C15—H15A	110.1	O10—C36—H36B	110.1
C16—C15—H15A	110.1	C37—C36—H36B	110.1
O3—C15—H15B	110.1	H36A—C36—H36B	108.4
C16—C15—H15B	110.1	O11—C37—C36	111.95 (10)
H15A—C15—H15B	108.4	O11—C37—H37A	109.2
O4—C16—C15	107.35 (9)	C36—C37—H37A	109.2
O4—C16—H16A	110.2	O11—C37—H37B	109.2
C15—C16—H16A	110.2	C36—C37—H37B	109.2
O4—C16—H16B	110.2	H37A—C37—H37B	107.9
C15—C16—H16B	110.2	O11—C38—C39	125.83 (11)
H16A—C16—H16B	108.5	O11—C38—C43	115.04 (10)
O4—C17—C18	114.69 (10)	C39—C38—C43	119.12 (11)
O4—C17—H17A	108.6	C38—C39—C40	119.85 (11)
C18—C17—H17A	108.6	C38—C39—H39A	120.1
O4—C17—H17B	108.6	C40—C39—H39A	120.1
C18—C17—H17B	108.6	C41—C40—C39	120.63 (11)
H17A—C17—H17B	107.6	C41—C40—H40A	119.7
O5—C18—C17	107.36 (10)	C39—C40—H40A	119.7
O5—C18—H18A	110.2	C40—C41—C42	120.11 (12)
C17—C18—H18A	110.2	C40—C41—H41A	119.9
O5—C18—H18B	110.2	C42—C41—H41A	119.9
C17—C18—H18B	110.2	C43—C42—C41	119.65 (11)
H18A—C18—H18B	108.5	C43—C42—H42A	120.2
O5—C19—C20	125.28 (10)	C41—C42—H42A	120.2
O5—C19—C24	115.47 (10)	C42—C43—O12	123.61 (10)
C20—C19—C24	119.25 (10)	C42—C43—C38	120.61 (10)
C21—C20—C19	119.61 (11)	O12—C43—C38	115.72 (10)
			
C5—N1—C1—C2	−0.47 (15)	C25—O6—C24—C19	78.83 (12)
C5—N1—C1—C25	179.56 (9)	O5—C19—C24—C23	177.31 (11)
N1—C1—C2—C3	−1.01 (17)	C20—C19—C24—C23	−2.15 (17)
C25—C1—C2—C3	178.96 (10)	O5—C19—C24—O6	−5.36 (15)
C1—C2—C3—C4	0.88 (17)	C20—C19—C24—O6	175.18 (10)
C2—C3—C4—C5	0.65 (17)	C24—O6—C25—O7	72.70 (11)
C1—N1—C5—C4	2.15 (15)	C24—O6—C25—C1	−169.87 (9)
C1—N1—C5—C6	−176.03 (9)	C26—O7—C25—O6	−123.97 (9)
C3—C4—C5—N1	−2.26 (16)	C26—O7—C25—C1	120.53 (10)
C3—C4—C5—C6	175.76 (10)	N1—C1—C25—O6	103.06 (10)
C43—O12—C6—O1	90.74 (11)	C2—C1—C25—O6	−76.92 (12)
C43—O12—C6—C5	−152.80 (9)	N1—C1—C25—O7	−140.77 (9)
C7—O1—C6—O12	−56.35 (11)	C2—C1—C25—O7	39.25 (13)
C7—O1—C6—C5	−172.01 (8)	C25—O7—C26—C27	133.16 (11)
N1—C5—C6—O12	62.05 (11)	C25—O7—C26—C31	−51.29 (14)
C4—C5—C6—O12	−116.15 (11)	O7—C26—C27—C28	176.45 (12)
N1—C5—C6—O1	−179.39 (8)	C31—C26—C27—C28	0.77 (19)
C4—C5—C6—O1	2.42 (13)	C26—C27—C28—C29	0.7 (2)
C6—O1—C7—C8	111.11 (11)	C27—C28—C29—C30	−1.1 (2)
C6—O1—C7—C12	−72.60 (13)	C28—C29—C30—C31	0.2 (2)
O1—C7—C8—C9	175.50 (10)	C32—O8—C31—C30	−60.17 (14)
C12—C7—C8—C9	−0.77 (17)	C32—O8—C31—C26	123.61 (11)
C7—C8—C9—C10	1.06 (18)	C29—C30—C31—O8	−174.92 (11)
C8—C9—C10—C11	0.0 (2)	C29—C30—C31—C26	1.23 (18)
C9—C10—C11—C12	−1.42 (19)	O7—C26—C31—O8	−0.86 (16)
C13—O2—C12—C11	−22.83 (16)	C27—C26—C31—O8	174.58 (11)
C13—O2—C12—C7	159.87 (10)	O7—C26—C31—C30	−177.15 (10)
C10—C11—C12—O2	−175.52 (11)	C27—C26—C31—C30	−1.71 (17)
C10—C11—C12—C7	1.68 (17)	C31—O8—C32—C33	119.02 (11)
C8—C7—C12—O2	176.86 (10)	C34—O9—C33—C32	−161.94 (10)
O1—C7—C12—O2	0.65 (15)	O8—C32—C33—O9	−75.05 (12)
C8—C7—C12—C11	−0.60 (16)	C33—O9—C34—C35	−80.07 (13)
O1—C7—C12—C11	−176.81 (10)	C36—O10—C35—C34	79.12 (13)
C12—O2—C13—C14	−165.98 (10)	O9—C34—C35—O10	70.00 (14)
C15—O3—C14—C13	−170.45 (10)	C35—O10—C36—C37	−175.63 (10)
O2—C13—C14—O3	59.66 (12)	C38—O11—C37—C36	78.96 (13)
C14—O3—C15—C16	−179.28 (9)	O10—C36—C37—O11	61.68 (13)
C17—O4—C16—C15	−169.69 (10)	C37—O11—C38—C39	8.08 (17)
O3—C15—C16—O4	71.86 (11)	C37—O11—C38—C43	−173.19 (10)
C16—O4—C17—C18	−65.62 (14)	O11—C38—C39—C40	177.70 (11)
C19—O5—C18—C17	173.18 (10)	C43—C38—C39—C40	−0.98 (17)
O4—C17—C18—O5	79.00 (13)	C38—C39—C40—C41	1.21 (19)
C18—O5—C19—C20	2.43 (17)	C39—C40—C41—C42	0.21 (19)
C18—O5—C19—C24	−176.99 (10)	C40—C41—C42—C43	−1.85 (18)
O5—C19—C20—C21	−177.06 (11)	C41—C42—C43—O12	179.04 (10)
C24—C19—C20—C21	2.34 (17)	C41—C42—C43—C38	2.08 (17)
C19—C20—C21—C22	−0.7 (2)	C6—O12—C43—C42	44.68 (14)
C20—C21—C22—C23	−1.1 (2)	C6—O12—C43—C38	−138.22 (10)
C21—C22—C23—C24	1.3 (2)	O11—C38—C43—C42	−179.49 (10)
C22—C23—C24—O6	−176.99 (12)	C39—C38—C43—C42	−0.67 (16)
C22—C23—C24—C19	0.35 (19)	O11—C38—C43—O12	3.32 (14)
C25—O6—C24—C23	−103.82 (12)	C39—C38—C43—O12	−177.86 (10)

### Hydrogen-bond geometry (Å, º)

Cg1 and Cg2 are the centroids of the C7–C12 benzene ring and the N1/C1–C5 pyridine ring, respectively.

**Table:**

*D*—H···*A*	*D*—H	H···*A*	*D*···*A*	*D*—H···*A*
C8—H8*A*···O4^i^	0.93	2.44	3.2871 (14)	151
C17—H17*A*···O11^ii^	0.97	2.60	3.4207 (15)	142
C3—H3*A*···*Cg*1^i^	0.93	2.94	3.6031 (15)	130
C18—H18*B*···*Cg*2^i^	0.97	2.80	3.7531 (15)	167

Symmetry codes: (i) *x*+1, *y*, *z*; (ii) *x*−1, *y*, *z*.
